# Structure-enabled enhancement of potency in a metronidazole–benznidazole hybrid: design, synthesis, and evaluation of antitrypanosomal activity of a benzylamide-linked 5-nitroimidazole

**DOI:** 10.3389/fphar.2026.1812023

**Published:** 2026-04-30

**Authors:** Afonso Santine M. M. Velez, Otávio Augusto Chaves, Telma Costa, Carlos Serpa, Joyce de Meyrelles Borges, Célio Geraldo Freire-de-Lima, Debora Decote-Ricardo, Marco Edilson Freire Lima

**Affiliations:** 1 Department of Organic Chemistry, Institute of Chemistry, Federal Rural University of Rio de Janeiro, Seropédica, Brazil; 2 Department of Chemistry, Coimbra Chemistry Centre-Institute of Molecular Science, University of Coimbra, Coimbra, Portugal; 3 Laboratory of Immunopharmacology, Center for Research, Innovation and Surveillance in COVID-19 and Health Emergencies (CPIV), Oswaldo Cruz Institute (IOC), Oswaldo Cruz Foundation (FIOCRUZ), Rio de Janeiro, Brazil; 4 Departamento de Microbiologia e Imunologia Veterinária, Instituto de Veterinária, Universidade Federal Rural do Rio de Janeiro, Seropédica, Brazil; 5 Instituto de Biofísica Carlos Chagas Filho, Universidade Federal do Rio de Janeiro, Rio de Janeiro, Brazil

**Keywords:** antiparasitic drugs, Chagas disease, drug design, molecular hybridization, nitroimidazole, *Trypanosoma cruzi*

## Abstract

Benznidazole (**1**) remains the primary antiparasitic drug used clinically for Chagas disease, underscoring the need to discover novel treatments targeting *Trypanosoma cruzi* (*T. cruzi*) infection. In this sense, the present work reports the *in silico* design of a novel hybrid (**3**) containing the 5-nitroimidazole core from the commercial antiparasitic drug metronidazole (**2**) and the *N*-benzylacetamide moiety from **1**. These structural motifs were designed to target *T. cruzi* nitroreductase type I (TcNTR) for activation and to boost intracellular drug levels (lipophilicity). The *in silico* predictions suggested that the presence of the *N*-benzylacetamide moiety might enhance the anti-T*. cruzi* activity of **2** and improve the drug-likeness. To validate the predictions, hybrid **3** was synthesized with a yield of 53% and structurally characterized (melting point, ^1^H and ^13^C NMR, and HRMS). The *in vitro* assays of hybrid **3** against *T. cruzi* amastigotes (Tulahuen strain C2C4 *LacZ*) supported the *in silico* predictions, showing a lower IC_50_ for **3** (67.73 ± 8.98 µM) than for **2** (>100 µM). Despite hybrid **3** having an activity 45-fold lower than that of **1**, the results provide insights for future hybrid optimization.

## 1 Introduction

**FIGURE 1:**
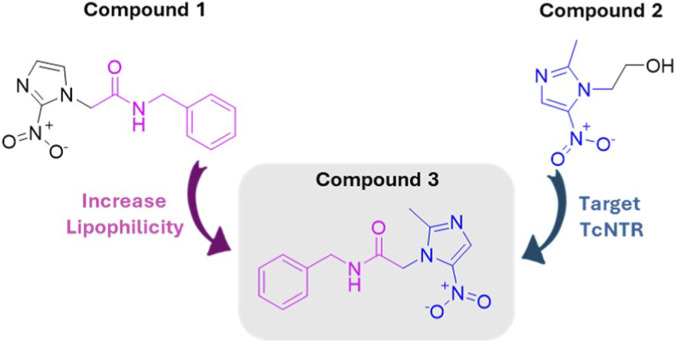
Chemical structure for benznidazole (**1**), metronidazole (**2**), and *N*-benzyl-2-(2-methyl-5-nitro-1*H*-imidazol-1-yl) acetamide derivative (**3**).

Nitroimidazoles are pharmacophoric groups widely used in drugs to treat parasitic infections (Patterson and Wyllie, 2014). Benznidazole (compound **1**, Figure 1) is an example of a clinically used nitroimidazole to treat Chagas disease, a chronic infection caused by *Trypanosoma cruzi* (*T. cruzi*), which results in around 10,000 to 12,000 deaths annually, primarily from heart failure or sudden cardiac death (Pérez-Molina and Molina, 2018). Another member of this class is metronidazole (compound **2**, Figure 1), which is used to treat infections caused by the protozoa *Giardia lamblia* and *Trichomonas vaginalis* (Cosar and Julou, 1959; Leitsch et al., 2012). A notable difference between these two drugs is the position of the nitro group: 2-nitroimidazole in benznidazole and 5-nitroimidazole in metronidazole. The effect of the position of the nitro group in the imidazole core was previously assessed in terms to impact the antiparasitic and cytotoxicity profile, e.g., Sánchez-Pavón and coworkers (Sánches-Pavón et al., 2012) reported that the mixture of isomers nitro 4(5)-bromo-1-phenacyl-5(4)-nitroimidazole derivatives is a feasible and potent therapeutic approach against *T. cruzi*, and Buschini and coworkers (Buschini et al., 2007) reported that the 5-nitromegazol might induce oxidative stress to achieve DNA damage (genotoxicity in the host cells), while 4-nitromegazol did not produce any significant effect, which indicated the relevance of the nitro position to decrease the toxicity to the host cell.

Unfortunately, the currently available treatment for Chagas disease is primarily based on a single drug, the compound **1**. In a few countries, nifurtimox (Lampit®, a 5-nitrofuran derivative) is prescribed to treat the acute phase of Chagas disease (Altcheh et al., 2025). Thus, different reports are searching novel antiparasitic candidates based on the derivatization approach of the pharmacophoric nitroimidazole or nitrofuran core (Maya et al., 2003; Rodríguez et al., 2025), as well as via the bioisosterism approach, as reported to 2-nitroimidazole-*N*-acylhydrazones derivatives that had half-maximal inhibitory concentration (IC_50_) in the range of 4.3–6.25 µM (compared to benznidazole, which demonstrated an IC_50_ of 1.52 μM within the same testing protocol) and proved to be highly selective with low cytotoxicity on L929 cells (cultured mouse fibroblast cell line), being considered feasible substrate to the enzyme *T. cruzi* nitroreductase type I (TcNTR) (Pitombeira et al., 2024). Additionally, drug hybrids containing a nitroimidazole core were reported to boost efficacy against parasite stages, reduce toxicity, and overcome resistance (Gonçalves-Santos et al., 2023). As an example, the eugenol-benznidazole hybrid reported by de Melo and coworkers (de Melo et al., 2025) was more active than benznidazole against both trypomastigote (IC_50_ of 1.8 and 6.1 µM, respectively) and amastigote forms (IC_50_ of 1.6 and 3.1 µM, respectively), also targeting TcNTR. In these two studies, BZN was used as a reference drug, exhibiting an IC_50_ of 5.14-6.1 µM against *T. cruzi* trypomastigotes (Y strain).

As highlighted above, TcNTR plays a crucial role in activating the nitroimidazole pharmacophore, leading to the formation of reductive metabolites that may cause deleterious effects on the parasite, including DNA damage and thiol depletion (Cirqueira et al., 2022). Thus, this enzyme also catalyzes the activation of the clinically used prodrug **1** via a ping-pong mechanism (Hall and Wilkinson, 2012). Recently, our group designed a series of dimeric 2-nitroimidazoles based on their *in silico* interactions with TcNTR (Velez et al., 2026). The cell-based assays validated *in silico* predictions, identifying longer-chain dimers with remarkable potency (IC50 < 1.0 µM) and demonstrating that targeting TcNTR is a promising strategy for designing novel antiparasitic compounds.

In this sense, to continue to contribute to the development of anti-*T. cruzi* candidates, the present work reports *in silico* design of a novel hybrid between the two commercial antiparasitic compounds **1** (benznidazole, considering its lipophilic *N*-benzylacetamide moiety) and **2** (considering its nitroimidazole to target TcNTR), namely *N*-benzyl-2-(2-methyl-5-nitro-1*H*-imidazol-1-yl) acetamide derivative (metronidazole, compound **3**, Figure 1) by molecular docking calculations with TcNTR combined with physicochemical properties and drug-likeness predictions. Molecular hybridization is a widely used approach in medicinal chemistry where structural fragments from different bioactive molecules are combined into a single scaffold to develop compounds with improved pharmacological properties (Murteira Pinheiro et al., 2024).

Metronidazole (**2**), a 5-nitroimidazole derivative, has been used for decades to treat anaerobic bacterial infections and intestinal protozoan diseases, with a well-established safety and tolerability profile (Hernández Ceruelos et al., 2019). In contrast, benznidazole (**1**), a 2-nitroimidazole, remains one of the few therapeutic options for Chagas disease but is associated with frequent adverse effects that limit long-term treatment. From a medicinal chemistry perspective, these contrasting profiles highlight the potential of clinically validated nitroimidazole scaffolds as starting points for rational structural modification to confer antitrypanosomal activity while preserving favorable safety features. In this way, the hybrid **3** was synthesized and structurally characterized to validate theoretical predictions, using cell-based assays against *T. cruzi* amastigotes (Tulahuen C2C4 *LacZ*) and cytotoxicity assays in an epithelial cell line derived from rhesus monkey kidney tissue (*Macaca mulatta*, LLC-MK2). To the best of our knowledge, the benzylamide derivative of the 2-methyl-5-nitroimidazole nucleus has not been reported in the peer-reviewed literature. Its only prior mention appears in a patent (Goldfarb, 2009), where it is listed by name without structural or characterization data, and in an academic dissertation (Costa, 2018), which is limited to demonstrating synthetic feasibility using a route distinct from that adopted in the present work. Therefore, the current study ought to be regarded not solely as a report on chemical synthesis but rather as a thorough investigation in medicinal chemistry that encompasses design, biological assessment, and mechanistic analysis.

## 2 Results and discussion

### 2.1 *In silico* hybrid design as a feasible antiparasitic drug

To reduce time and cost, *in silico* tools are widely used as initial approaches in drug design and discovery. In this sense, molecular docking calculations are used to predict interactions, design novel molecules, repurpose existing drugs, and understand mechanisms (Roney and Mohd Aluwi, 2024; Chaves et al., 2025). Thus, as a first step toward identifying potential molecular hybrids based on commercial compounds against *T. cruzi*, molecular docking calculations were performed for compounds **1-3**. Since the two commercial compounds **1** and **2** and the proposed hybrid **3** share a nitroimidazole core, their theoretical interaction profiles with TcNTR were explored using the reported three-dimensional structure in the presence of the coenzyme flavin mononucleotide (FMN), which has no homolog in the human host (Velez et al., 2026).

In the GOLD 2025 software, the docking score value (dimensionless) for each pose accounts for intramolecular tensions within the ligand and intermolecular interactions and is considered as the negative value of the sum of the energy terms involved in the macromolecule–ligand association; thus, the more positive the score, the better the interactive profile (Machado et al., 2025). The obtained docking score values were 50.7, 44.3, and 48.9 for TcNTR:**1**, TcNTR:**2**, and TcNTR:**3**, respectively, highlighting that the commercial drug benznidazole still might present the best interactive profile with TcNTR to be activated; however, the hybrid **3** increased the capacity of compound **2** to interact with TcNTR, suggesting a better antiparasitic profile than the commercial metronidazole. It is important to highlight that for each ligand, a total of 10 poses were obtained in the evaluated enzymatic structure and Supplementary Figure S1 in the Supplementary Material shows the superposition of these poses, demonstrating the high reproducibility of the molecular docking trend, which is reinforced by the similarity in the docking score values, i.e., TcNTR:**1** are in the range of 50.7–43.9, while for TcNTR:**2** are 44.3–41.7, and for TcNTR:**3** are 43.1–48.9.

**FIGURE 2:**
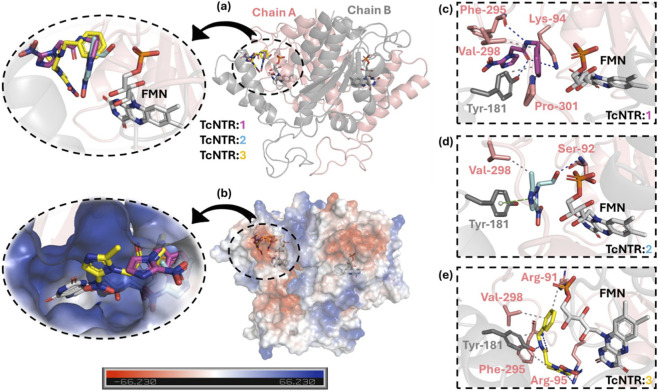
**(a)** Superposition of the best docking pose of the interaction TcNTR:**1-3** into the active site of the enzyme. **(b)** The electrostatic potential map of TcNTR in the presence of compounds **1-3**. The main amino acid residues from TcNTR interact with compounds **(c) 1**, **(d) 2**, and **(e) 3** in the presence of coenzyme FMN. Black and green dots mean hydrophobic and π-stacking interactions, while the blue line means a hydrogen bond. The amino acid residues from chains A and B, FMN, and the compounds **1, 2,** and **3** are depicted as sticks in beige, black, white, pink, cyan, and yellow, respectively. Elements’ color: oxygen, phosphorus, and nitrogen in red, orange, and blue, respectively. For better interpretation, hydrogen atoms were omitted.

Figure 2 depicts the docking pose for TcNTR:**1-3** in the catalytic pocket, highlighting the difference in the pose of compound **2** compared with **1** and **3** (Figure 2a), i.e., the *N*-benzylacetamide moiety of compounds **1** and **3** were in a similar pose in chain A of the enzyme, suggesting that metronidazole will have the most different antiparasitic capacity. Since compounds **1-3** have delocalized electrons and electronegative atoms, they were well buried in a positive electrostatic pocket of TcNTR (Figure 2b), being stabilized mainly by hydrogen bonds and hydrophobic forces (Figures 2c–e; Table 1); however, a π-stacking interaction was also detected to TcNTR:**2** within a distance of 3.92 Å. Finally, the number of connecting points to TcNTR:**2** is very low compared with TcNTR:**1** and TcNTR:**3** (Figures 2c–e; Table 1), suggesting that the presence of the *N*-benzylacetamide moiety in the metronidazole core might increase its antiparasitic capacity.

**TABLE 1:** The main amino acid residues and the corresponding interactive forces for the interaction TcNTR:**1**-**3**.

Compound	Amino acid residues	Interaction	Distance (Å)
**1**	Lys-94	Hydrogen bond	3.91
	Tyr-181	Hydrophobic	3.63
	Tyr-181	Hydrogen bond	1.63
	Phe-295 (Peptidic bond)	Hydrogen bond	3.01
	Val-298	Hydrophobic	3.45
	Pro-301	Hydrophobic	3.67
**2**	Ser-92 (Peptidic bond)	Hydrogen bond	2.10
	Tyr-181	π-stacking	3.92
	Val-298	Hydrophobic	3.58
**3**	Arg-91	Hydrophobic	3.64
	Arg-95	Hydrogen bond	2.93
	Tyr-181	Hydrophobic	3.58
	Tyr-181	Hydrogen bond	1.63
	Phe-295	Hydrophobic	3.14
	Phe-295 (Peptidic bond)	Hydrogen bond	3.04
	Val-298	Hydrophobic	3.67

**FIGURE 3:**
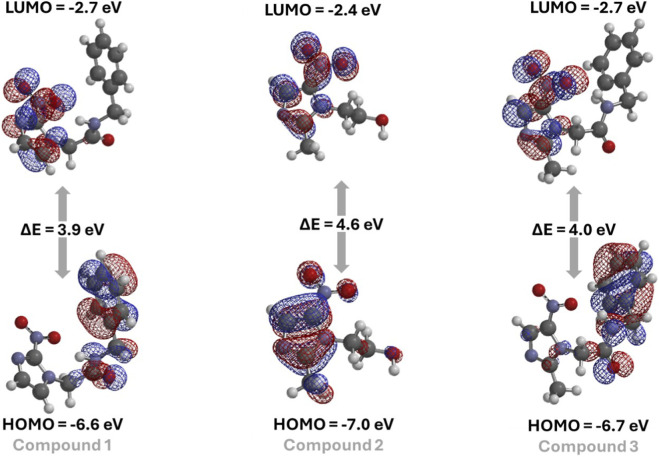
The FMO densities and corresponding energetic values for compounds **1-3**. Parameters for calculation: DFT-B3LYP/6-31G*.

One indicator of reactivity between small molecules and protein cavities is the energy of the frontier molecular orbitals (FMOs), specifically the highest occupied molecular orbital (HOMO) and the lowest unoccupied molecular orbital (LUMO). In this case, compounds with the lowest HOMO-LUMO energy gap are readily reactive (Martins et al., 2025; Siqueira et al., 2026). Figure 3 depicts the FMOs density with the corresponding HOMO, LUMO, and HOMO-LUMO energy gap to the compounds **1-3**. Since compound **2** had the highest HOMO-LUMO energy gap, it indicates the lowest ability to transfer electrons in a redox reaction, suggesting that compounds **1** and **3** have the highest potential to act as antiparasitic drugs, supporting the molecular docking trend. Finally, the LUMO density is localized in the nitroimidazole core, where the nitro group is present, reinforcing the capacity of the evaluated compounds to undergo reduction within the catalytic pocket of TcNTR.

In addition to the *in silico* antiparasitic profile of compounds **1-3**, the preliminary pharmacokinetic properties and drug-likeness of the hybrid were predicted using the free web server SwissADME (Daina et al., 2017). The same properties were also predicted for the two commercial compounds **1** and **2** (Supplementary Figures S2-S4 in the Supplementary Material), to compare the trend. As expected, the presence of the *N*-benzylacetamide moiety in the hybrid increased the consensus lipophilicity (log P_o/w_) relative to compound **2** (Table 2), potentially enhancing its intracellular concentration. Despite the difference in the nitro position and presence of a methyl group in the nitroimidazole core of the hybrid, the compounds **3** and **1** had the same surface contributions of polar functional groups as evidenced by their topological polar surface area values (TPSA, Table 2), suggesting the similarity in the drug transport properties, such as intestinal absorption, and blood-brain barrier penetration (Prasanna and Doerksen, 2009). The predicted differences in the adsorption, distribution, metabolism, and excretion (ADME) of the compounds **1** and **2** (Supplementary Figures S2 and S3 in the Supplementary Material) agree with their reported experimental differences in doses, terminal half-life, and median maximum concentration (Ralph, 1983; Molina et al., 2017; Assmus et al., 2025; Shahzad et al., 2025); however, since the predicted parameters of compounds **3** is similar to those obtained for **1** (Supplementary Figures S2 and S4 in the Supplementary Material), suggest that the hybrid might present similar ADME characteristics reported to benznidazole.

**TABLE 2:** Selected physicochemical, drug-likeness, and pharmacokinetic properties predicted by the free web server SwissADME (Daina et al., 2017).

Compound	Consensus Log P_o/w_ ^a^	TPSA (Å^2^)	Drug-likeness (number of violations)				
			Lipinski	Ghose	Veber	Egan	Muegge
**1**	0.49	92.74	Yes (0)	Yes (0)	Yes (0)	Yes (0)	Yes (0)
**2**	−0.32	83.87	Yes (0)	Yes (0)	Yes (0)	Yes (0)	No (1)^b^
**3**	0.78	92.74	Yes (0)	Yes (0)	Yes (0)	Yes (0)	Yes (0)

^a^ Consensus log P_o/w_: octanol/water partition coefficient calculated as the mean of five distinct predictive log P methods.

^b^ Molecular weight <200 g/mol.

Finally, the three compounds under study had potential drug-likeness, which was reinforced by the non-violations in the five drug-likeness approximations (Lipinski, Ghose, Veber, Egan, and Muegge; Table 2), except for compound **2** in the Muegge criteria, which had a molecular weight of less than 200 g/mol, indicating that the suggested hybrid might have more capacity to act as a feasible drug than the commercial compound metronidazole. It is important to recognize that the *in silico* data obtained is a first proof-of-concept study on the binding of TcNTR:**1-3**. Thus, future combination of biochemical and biophysical assays, such as determining the enzymatic inhibitory mechanism, performing target engagement and binding assays to measure the strength of interactions, and structural experiments, should be carried out to further understand how compounds **1-3** target TcNTR and their drug-likeness properties (Rocha et al., 2026). However, in this study, as an initial step to support the *in silico* antiparasitic profile, the hybrid was synthesized, and *in vitro* antiparasitic assays were performed on compounds **1**-**3**, along with the determination of their electrochemical profiles.

### 2.2 Chemistry

**SCHEME 1:**
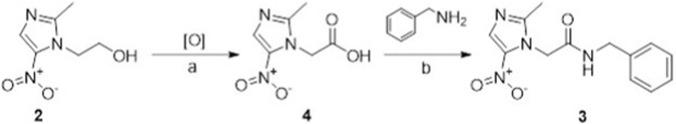
Synthesis of hybrid (**3**) based on the commercial benznidazole and metronidazole moieties. Reaction conditions: (a) propanone, H_2_CrO_4_ aq. (55% yield); (b) HCTU, HOBT, DIPEA, benzylamine, DMF (53% yield).

Although metronidazole itself is inactive against *T. cruzi* amastigotes (Lopes et al., 2011), its excellent clinical tolerability makes the 5-nitroimidazole core an attractive scaffold for structural re-engineering rather than direct drug repositioning. A classical oxidation reaction was conducted using an aqueous chromic acid solution (Jones’ reagent) (Chandrasekaran and Ganesh, 2014) and the commercial drug metronidazole as the starting material. Under these conditions, the carbinolic carbon is oxidized directly to the corresponding carboxylic acid, as the intermediate formed aldehyde is hydrated by the action of the acidic aqueous medium, resulting in the hydrate of the aldehyde, which is immediately converted to the carboxylic acid by the action of the Cr^+6^ reagent. Under these reaction conditions, the respective acid was obtained in 55% yield. We used the coupling reagent *O*-(1H-6-chlorobenzotriazol-1-yl)-1,1,3,3-tetramethyluronium hexafluorophosphate (HCTU) and *N*-Hydroxybenzotriazole (HOBt) in *N*,*N*-dimethylformamide (DMF) in the presence of *N*,*N*-diisopropylethylamine (DIPEA) for the amide formation step (Hood et al., 2008). Upon prior activation of the carboxyl group, the acid reacts with benzylamine, yielding the desired product in 53% yield (Scheme 1).

The synthetic strategy adopted in this study was deliberately designed to enhance the functional profile of the otherwise poorly active 5-nitroimidazole scaffold of metronidazole by installing the benzylamide motif characteristic of benznidazole. Oxidation of the primary alcohol to the corresponding carboxylic acid furnished a chemically robust and strategically valuable intermediate, enabling late-stage amide formation while preserving the redox-active nitroimidazole core. Although amide coupling was performed using a specific coupling protocol (HCTU, HOBT, DIPEA) (Hood et al., 2008), this step is inherently modular and can be readily achieved through alternative carboxyl-activation protocols, including carbodiimide-mediated coupling, activated esters, mixed anhydrides, or CDI-based approaches (Montalbetti and Falque, 2005; Valeur and Bradley, 2009), thereby enabling systematic SAR exploration of amide substituents and linker architectures. Hybrid **3** was comprehensively characterized using standard organic analytical techniques, and the resulting spectra are presented in the Supplementary Material (Supplementary Figures S5-S10). Consistent with this rationale, docking analyses indicate that the hybrid adopts a TcNTR-binding orientation and a hydrogen-bonding network that more closely resembles those of benznidazole than of metronidazole, highlighting the amide-linked aromatic fragment as a key determinant of productive molecular recognition. In parallel, ADME predictions indicate that incorporating the benzylamide unit shifts the hybrid’s physicochemical profile toward a benznidazole-like space, particularly in terms of lipophilicity and polar surface balance.

### 2.3 Biological assays

To validate the *in silico* predictions of the design of a novel hybrid as a feasible antiparasitic inhibitor, compound **3** was assayed against *T. cruzi* amastigotes (Tulahuen C2C4-*LacZ* strain). Given that *T. cruzi* replicates within mammalian cells, thereby driving disease progression (leading to Chagas disease), the amastigote form of *T. cruzi* was used instead of the epimastigote form (Buckner et al., 1996). The commercial compounds **1** and **2** were also evaluated, and the resulting IC_50_ and 50% cytotoxic concentration (CC_50_) values, along with the corresponding selective index (SI), are summarized in Table 3.

**TABLE 3:** The biological activity of compounds **1-3** in terms of inhibition of *T. cruzi* amastigotes (IC_50_), cytotoxicity (CC_50_), and selective index (SI).

Compound	Activity against amastigotes of *T. cruzi* (tulahuen C2C4 lacZ) IC_50_ (µM)	Cytotoxicity in LLC-MK2 CC_50_ (µM)	SI^a^
**1** ^b^	1.50 ± 0.33	>200	>133.3
**2**	>100	>200	n.d.^c^
**3**	67.73 ± 8.98	>200	>2.95

^a^ SI, Selectivity index = (CC_50_ LLC-MK2)/(IC_50_ *T. cruzi*).

^b^ Reference drug.

^c^ n.d., not determined.

All evaluated compounds had low cytotoxicity against LLC-MK2 cells (CC_50_ > 200 µM); however, the reference drug (**1**) is still the most potent with an IC_50_ value of 1.50 ± 0.33 µM, agreeing with the *in silico* highest docking score value for TcNTR and the predicted lowest HOMO-LUMO energy gap. Despite the hybrid **3** being 45-fold less active than benznidazole, this result provides structural insights for future optimization, indicating that the position of the nitro group (from 5- to 2-nitroimidazole) or the absence of a methyl group is relevant to achieving an efficient antiparasitic profile and SI value.

Additionally, cell-based assays corroborated the *in silico* trend, showing that the designed hybrid **3** has a better antiparasitic profile than the commercial metronidazole **2**, with IC_50_ values of 67.73 ± 8.98 and >100 µM for compounds **3** and **2**, respectively (Table 3). This improvement in biological activity demonstrates that the implemented structural changes produced a moderately active derivative against intracellular amastigotes of *T. cruzi*. The inhibition plots for the compounds are shown in Supplementary Figures S11, S12 in the Supplementary Material. These observations may be related to secondary factors, such as differences in physicochemical properties (e.g., lipophilicity and HOMO-LUMO energy gap), thereby facilitating the entry of hybrid **3** into parasite cells and its tendency to undergo activation at the catalytic site of TcNTR.

### 2.4 Cyclic voltammetry (CV) analysis

**FIGURE 4:**
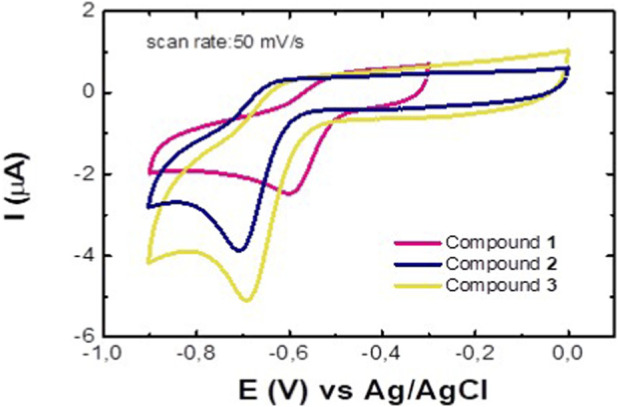
Cyclic voltammograms of compounds **1**, **2**, and **3** at pH 7, obtained in a saturated N_2_ environment at a scan rate of 50 mV/s.

Since nitroimidazoles are reduced by TcNTR to achieve their antiparasitic activity, CV measurements were carried out for the compounds **1-3** to further comprehend their antiparasitic profile. Figure 4 depicts the cyclic voltammograms for each compound in a 1 M KCl solution (pH 7). The respective voltammograms show irreversible cathodic peaks at −0.602 V, −0.708 V, and −0.694 V (vs. Ag/AgCl) for compounds **1**, **2**, and **3**, respectively. These peaks correspond to the reduction of the nitro group to the hydroxylamine derivative via a four-proton, four-electron process (Equation 1):

$$R-{\text{NO}}_{2}+4e^{-}+4\;H^{+}\;\to \;R-\text{NHOH}+H_{2}O$$ (1)

This reduction pathway is consistent with previous reports on the commercial drugs benznidazole (La-Scalea et al., 2002) and metronidazole under neutral and alkaline conditions (Ammar et al., 2016), as well as for other nitroimidazole derivatives (Brito et al., 2022), which achieve their antiparasitic activity via this pathway. Replacing the hydroxyl group from **2** with an *N*-benzylacetamide moiety had a slight effect on the cathodic peak potential, suggesting that the steric and electronic effects of this more external substituent did not significantly affect the electron density at the nitro group, as previously suggested via *in silico* HOMO-LUMO density. On the other hand, replacing 5-nitroimidazole with 2-nitroimidazole (comparing compounds **3** and **1**, respectively) shifted the reduction potential towards less negative values, indicating that the nitro position and removing the electron-donating groups (methyl group in compound **3**) on the heterocycle stabilizes the nitro group and increases its reduction potential, supporting the structural insights discussed in Section 2.3 for future optimization of the hybrid **3**.

**FIGURE 5:**
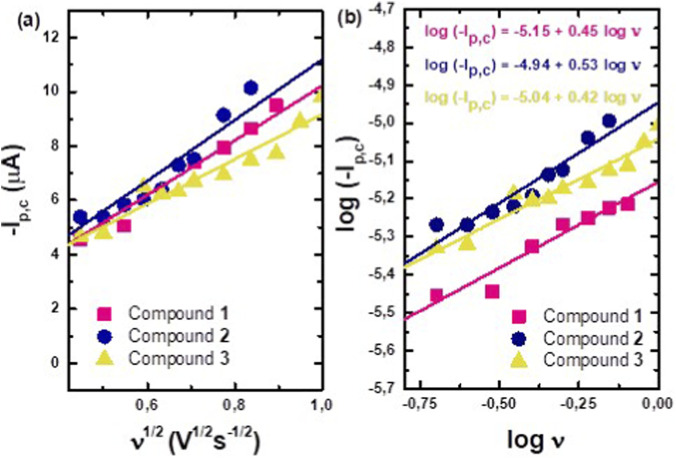
Plots of **(a)** I_p,c_ vs. the square root of the scan rate and **(b)** log I_c,p_ vs. log scan rate in the 200–800 mV/s scan rate range for compounds **1**, **2**, and **3** in a 1 M KCl aqueous solution.

The scan rate (ν) varied from 200 to 800 mV/s (Supplementary Figure S13 in the Supplementary Material), and a linear relationship was found between the reduction peak current intensity amplitude (I_p,c_) and the square root of the scan rate (Figure 5a), which is consistent with a diffusion-controlled process. This conclusion is further supported by the log-log plot of I_p,c_ vs. ν, which yields slopes ranging from 0.42 to 0.53 across the three systems studied (Figure 5b). These obtained values are close to the theoretical value of 0.5 at 25 °C (Gosser, 1993). The potential of the cathodic peak, E_p,c_, decreases by a value ranging from 40 to 57 mV as the scan rate increases (Supplementary Figure S14 in the Supplementary Material). Overall, the onset potential of the cathodic peak (E_red_, _onset_) for the compounds **1**, **2**, and **3** was observed at −0.49 V, −0.61 V, and −0.59 V (vs. Ag/AgCl), respectively, which correspond to the LUMO energies of −3.91, −3.79, and −3.81 eV, in the same trend obtained by physicochemical predictions (Section 2.1) that identified compound **2** with the lowest LUMO value.

## 3 Materials and methods

### 3.1 Chemistry

#### 3.1.1 Equipment, reagents, and solvents

Unless otherwise stated, all chemical reagents were purchased from Sigma–Aldrich (St. Louis, MO, USA). Solvents were treated with activated molecular sieves (3Å) before use. Reactions were monitored by thin-layer chromatography (TLC) on 0.25 mm Merck (Darmstadt, Germany) silica gel plates (60F-254) and visualized under a UV lamp (254 and 365 nm). All melting points (mp) were determined using a Fisatom 430D apparatus (São Paulo, Brazil) and were uncorrected. The proton and carbon nuclear magnetic resonance (^1^H-NMR and ^13^C-NMR, respectively) spectra were acquired on a Bruker Ultrashield Plus Spectrometer (BrukerBioSpin GmbH, Rheinstetten, Germany) operating at 500 MHz for ^1^H and 125 MHz for ^13^C at 25 °C, referenced to tetramethylsilane (TMS). Chemical shifts are reported in parts per million (ppm; *δ*) using the residual solvent line as an internal standard. Splitting patterns are designed as s, singlet; d, doublet; t, triplet; m, multiplet; brs, broad singlet. The liquid chromatography–mass spectrometry (LC-MS) analyses were performed using a Shimadzu LC-MS 2020 (Shimadzu Inc., Kyoto, Japan). Analytical conditions: column: Kromasil C18, 150 mm × 4.6 mm × 5 µm (AkzoNobel, Amsterdam, Netherland); mobile phase: water with 0.1% formic acid (A), acetonitrile with 0.1% formic acid (B), 1.0 mL/min, linear gradient (indicated on trace); injection volume: 10 µL; detectors: PDA (200–400 nm), ESI^+^ (low resolution). High-resolution mass spectrometry (HRMS) analyses were performed using a Bruker COMPACT QTOF (Bruker-Daltonics, Bremen, Germany).

#### 3.1.2 Synthesis of 2-(2-methyl-5-nitro-1*H*-imidazol-1-yl)acetic acid (4)

In a round-bottom flask equipped with a magnetic stirrer, 171 mg (1.0 mmol) of metronidazole was dissolved in 10 mL of acetone at room temperature. Then, 1 mL of Jones’ reagent (CrO_3_ in aqueous H_2_SO_4_) was added dropwise to the reaction mixture until a dark orange color was obtained. After 24 h of reaction, the mixture turned dark green, indicating that all reagents had been consumed. Subsequently, 10 mL of isopropyl alcohol was added to reduce Cr (VI) to Cr (III), ensuring that no Cr (VI) remained in the reaction. The reaction was filtered, dried over anhydrous sodium sulfate (Na_2_SO_4_), filtered again, and concentrated under reduced pressure in a rotary evaporator. The product was isolated as a white solid (102 mg, 0.55 mmol, 55% yield). Mp = 174 °C–176 °C. Structure was confirmed by ^1^H NMR, ^13^C NMR, and MS: ^1^H NMR (500 MHz, Acetone-*d*_*6*_): *δ* 7.95 (s, 1H); 5.23 (s, 2H); 2.51 (s, 3H). ^13^C NMR (125 MHz, Acetone-*d*_*6*_): *δ* 167.7; 131.7; 47.0; 13.0. All spectra were provided as Supplementary Figures S5, S6 in the Supplementary Material.

#### 3.1.3 Synthesis of *N*-benzyl-2-(2-methyl-5-nitro-1*H*-imidazol-1-yl)acetamide (3)

In a round bottom flask, equipped with a magnetic stirred, 20 mg (0.11 mmol) of **4**, 12 µL (0.11 mmol) of benzylamine, 45.5 mg (0.11 mmol) of HCTU, 14.8 mg (0.11 mmol) of hydroxybenzotriazole (HOBT), and 58 µL (0.33 mmol) of *N*,*N*-diisopropylethylamine was solved in 0.5 mL of DMF at room temperature. The reaction was monitored by TLC for 6 h. The reaction was added to a 100 mL beaker containing crushed ice. The precipitate was then vacuum-filtered and dried at room temperature. The product was isolated as a white solid (16 mg, 0.058 mM, 53% yield). Mp = 145 °C-147 °C. Structure was confirmed by ^1^H NMR, ^13^C NMR, and HRMS: ^1^H NMR (500 MHz, CDCl_3_): *δ* 7.98 (s, 1H); 7.37 (m, 3H); 7.27 (d, 1H); 6.26 (s, 1H); 4.95 (s, 2H); 4.48 (d, 2H); 2.53 (s, 3H). ^13^C NMR (125 MHz, CDCl_3_): *δ* 164.8; 151.5; 137.0; 132.9; 128.9; 127.7; 48.6; 44.0; 14.2. HRMS (EI, *m/z*) calcd. for C_13_H_14_N_4_NaO_3_^+^, 297.0963; found 297.0958. HPLC (Gradient; ACN:H_2_O (50%-95% ACN), 15 min; 0.1% TFA): r.t. = 5.28 min, purity = 98.3%. All data used to characterize the chemical structure of hybrid **3** were provided as Supplementary Figures S7-S10 in the Supplementary Material.

### 3.2 Biological assays

Mammalian cells were used to evaluate the cytotoxicity of the tested compounds. LLC-MK2 cells (ATCC®) were cultured in Dulbecco’s modified Eagle’s medium (DMEM) supplemented with 5% fetal bovine serum (FBS) and incubated at 37 °C (5% CO_2_), with successive passages every 4-5 days. Cells were dissociated from the monolayer by treatment with a solution containing 0.25% w/v trypsin and 0.04% EDTA.

Antiparasitic activity assays used the Tulahuen C2C4-LacZ strain of *T. cruzi*, evaluated in the amastigote form [8], cultivated by successive reinfections in a monolayer of LLC-MK2 cells in DMEM supplemented with 2% FBS and incubated at 37 °C (5% CO_2_). Trypomastigote forms were collected from the culture supernatant between the 5th and 10th day after infection and separated from non-adherent cells by differential centrifugation.

The viability percentage values were entered into GraphPad Prism 9, where statistical analyses were performed and graphs were generated. For the calculation of the 50% growth inhibitory concentration (IC_50_), the software’s standard nonlinear regression model was used to fit inhibitor concentration versus normalized response with a variable slope. The IC_50_ values were obtained by averaging at least three independent experiments. For statistical significance testing, a one-way ANOVA was used.

#### 3.2.1 Evaluation of cytotoxicity against LLC-MK2

In a transparent 96-well plate, a suspension of 1 × 10^4^ LLC-MK2 cells (ATCC) in DMEM supplemented with 2% FBS was added. Cells were incubated at 37 °C (5% CO_2_) for 20 h, then washed with PBS to remove nonadherent cells. Cells were treated with serial dilutions of the hybrids (100–6.4 µM) in triplicate, pre-diluted in DMEM supplemented with 2% FBS. Untreated controls, vehicle (0.2% v/v dimethyl sulfoxide-DMSO), and a blank (no added cells) were included in the experiment. After 120 h of incubation, the supernatant was removed, and the cell monolayer was washed with PBS. Then, the culture medium was renewed. 20 µL of 3.0 mM 3-(4,5-dimethylthiazol-2-yl)-2,5-diphenyltetrazolium bromide (MTT saline) was added, followed by incubation for another 1.5 h. The supernatant was removed, and the MTT formazan crystals were dissolved by adding 120 µL of DMSO per well. After incubation for 1.5 h in the dark at 37 °C to dissolve the MTT crystals, the absorbance at 570 nm was measured using a plate reader.

#### 3.2.2 Evaluation of trypanocidal activity against amastigote forms of *T. cruzi*

In a transparent 96-well plate, a suspension of 1 × 10^4^ LLC-MK2 cells (ATCC) in DMEM supplemented with 2% FBS was added. Cells were incubated at 37 °C (5% CO_2_) for 3 h to promote adhesion, then washed with PBS to remove non-adherent cells. A suspension containing 1.5 × 10^5^ trypomastigotes of the *T. cruzi* Tulahuen C2C4 *LacZ* strain was added to the cells, followed by incubation at 37 °C (5% CO_2_) for 20 h to establish infection. Non-internalized parasites were removed by three successive washes with PBS, followed by treatment with serial dilutions of the hybrids in triplicate (100–0.06 µM), pre-diluted in DMEM supplemented with 2% FBS. Untreated, vehicle (0.2% v/v DMSO), and blank (no added parasites) controls were included in the experiment. Benznidazole (**1**) was used as a positive control in serial dilution. After incubation for 5 days (120 h), 30 µL of a 0.5 mM solution of the chlorophenol red *β*-galactopyranoside (CPRG) substrate in PBS containing 0.9% v/v Igepal CA-630 was added. After incubation for 1.5 h, absorbance was measured at 570 nm using a plate reader.

### 3.3 Cyclic voltammetry (CV) measurements

The CV measurements were obtained using an Autolab potentiostat/galvanostat PGSTAT204, operated with NOVA 2.1 software. The setup consisted of a three-electrode system comprising a glassy carbon electrode (GCE, diameter = 3 mm) as the working electrode, a GC wire (diameter = 1.6 mm) as the counter electrode, and an Ag/AgCl electrode (3.0 M KCl) as the reference electrode. Before each experiment, the GCE was meticulously polished using aluminium oxide particles of 0.3 µm and 0.075 µm (Elgrishi et al., 2018). The electrode was then thoroughly rinsed with Milli-Q water and sonicated in a 50:50 (v/v) Milli-Q water-ethanol solution for 5 min. The CV curves for compounds **1-3** were obtained at 1.0 mM in 1.0 M KCl aqueous solutions at pH 7 (adjusted with HCl/NaOH and measured before and after the CV measurements). The solutions were previously degassed with nitrogen for 10 min, and the nitrogen atmosphere was maintained throughout the CV experiments. The scan rate varied from 50 to 800 mV s^-1^ at room temperature. The onset potential was determined using the tangent-intersection method.

### 3.4 Molecular docking procedure

The experimental 3D structure of TcNTR Tulahuen C2C4 *LacZ* in the presence of the coenzyme FMN is unavailable; therefore, the previously reported theoretical protein model (Velez et al., 2026) was used. The chemical structures of compounds **1**-**3** were built and minimized using Density Functional Theory (DFT) at the B3LYP/6-31G* level with Spartan'14 (Wavefunction, Inc., Irvine, CA, USA) (Shao et al., 2006). The same software and method were used to obtain the physicochemical properties of the compounds. The molecular docking calculations were performed using the GOLD 2025 software (Cambridge Crystallographic Data Center, Cambridge CB2 1EZ, UK) (Jones et al., 1997) at pH 7.4. A 6 Å radius around the coenzyme FMN was considered as a grid box, and the default scoring function ChemPLP was used in docking runs due to the reported lowest root mean square deviation (RMSD) values for crystallographic compounds in other nitroreductase enzymes (Velez et al., 2026). Additionally, ChemPLP was considered as the scoring function due to our previously reported redocking calculations involving the enzymatic cofactor FMN crystallized in the NTR structure from *E. coli* B (Velez et al., 2026). A total of 10 poses were obtained per ligand, and more than 50% of the obtained poses are highly superposed (Supplementary Figure S1 in the Supplementary Material), indicating the reliability and reproducibility of the *in silico* trend. The web server Protein-Ligand Interaction Profiler (PLIP) (Adasme et al., 2021) was used for the identification of protein-ligand interactions, and the figures of the docking poses for the largest docking score value were generated with PyMOL Molecular Graphics System 1.0 level software (Delano Scientific LLC software, Schrodinger, New York, NY, USA) (Yuan et al., 2017).

### 3.5 ADME prediction

The absorption, distribution, metabolism, and excretion (ADME) properties, along with physicochemical, drug-like, and related parameters, were estimated for the compounds using the free web server SwissADME (SwissADME, 2025.; Daina et al., 2017).

## 4 Conclusion

A novel hybrid (**3**) based on the commercial drugs benznidazole (**1**) and metronidazole (**2**) was designed targeting the positive electrostatic potential pocket of TcNTR to undergo activation. *In silico* trend suggested that the presence of the *N*-benzylacetamide moiety in compound **2** could improve its antiparasitic profile, as experimentally validated following the organic synthesis of hybrid **3**. The synthetic methodology described here for obtaining hybrid **3** is efficient and adaptable, yielding a satisfactory amount and high purity. The product was evaluated against *T. cruzi* amastigotes, yielding an IC_50_ value lower than that of compound **2**, supporting *in silico* predictions. Although the benznidazole–metronidazole hybrid **3** was less potent than benznidazole itself, the introduction of the benznidazole-derived benzylamide moiety into the otherwise inactive 5-nitroimidazole scaffold resulted in a clear gain of antitrypanosomal activity, converting metronidazole from inactive (>100 µM) to a compound with measurable activity against intracellular amastigotes (EC_50_ = 67 µM). Collectively, these convergent synthetic, computational, and biological findings support a structure-based enhancement of antitrypanosomal activity relative to metronidazole, establishing this hybrid scaffold as a rational foundation for developing next-generation analogues to optimize the balance between potency and selectivity.

## Data Availability

The original contributions presented in the study are included in the article/Supplementary Material, further inquiries can be directed to the corresponding authors.

## References

[B1] Adasme M. F. Linnemann K. L. Bolz S. N. Kaiser F. Salentin S. Haupt V. J. (2021). PLIP 2021: expanding the scope of the protein–ligand interaction profiler to DNA and RNA. Nucleic Acids Res. 49, W530–W534. 10.1093/nar/gkab294 33950214 PMC8262720

[B2] Altcheh J. Grossmann U. Stass H. Springsklee M. Garcia-Bournissen F. (2025). Redefining the treatment of chagas disease: a review of recent clinical and pharmacological data for a novel formulation of nifurtimox. PLoS Negl. Trop. Dis. 19, e0012849. 10.1371/journal.pntd.0012849 39999088 PMC11856279

[B46] Ammar H. B. Brahim M. B. Abdelhédi R. Samet Y. (2016). Green electrochemical process for metronidazole degradation at BDD anode in aqueous solutions via direct and indirect oxidation. Sep. Purif. Technol. 157, 9–16. 10.1016/j.seppur.2015.11.027

[B3] Assmus F. Adehin A. Hoglund R. M. Fortes Francisco A. Lewis M. D. Kelly J. M. (2025). Pharmacokinetic-pharmacodynamic modeling of benznidazole and its antitrypanosomal activity in a murine model of chronic chagas disease. PLoS Negl. Trop. Dis. 19, e0012968. 10.1371/journal.pntd.0012968 40359193 PMC12074391

[B4] Brito C. L. Lins R. S. O. Bertotti M. Ferreira E. I. La-Scalea M. A. (2022). Free radical formation evidence from nimorazole electrochemical reduction in aqueous media. Electrochim. Acta 403, 139709. 10.1016/j.electacta.2021.139709

[B5] Buckner F. S. Verlinde C. L. La Flamme A. C. Van Voorhis W. C. (1996). Efficient technique for screening drugs for activity against Trypanosoma cruzi using parasites expressing beta-galactosidase. Antimicrob. Agents Chemother. 40 (11), 2592–2597. 10.1128/aac.40.11.2592 8913471 PMC163582

[B6] Buschini A. Giordani F. de Albuquerque C. N. Pellacani C. Pelosi G. Rossi C. (2007). Trypanocidal nitroimidazole derivatives: relationships among chemical structure and genotoxic activity. Biochem. Pharmacol. 73, 1537–1547. 10.1016/j.bcp.2007.01.024 17291457

[B7] Chandrasekaran S. Ganesh V. (2014). “7.10 oxidation adjacent to oxygen of alcohols by chromium reagents,” in Comprehensive organic synthesis II (Elsevier), 277–294. 10.1016/B978-0-08-097742-3.00711-4

[B8] Chaves O. A. Echevarria‐Lima J. Serpa C. Echevarria Á. (2025). Antitumoral profile of 1,3,4‐Thiadiazolium salts: insights into a combinatory therapeutic approach with flavonoid quercetin. Chem. Biodivers. 22, e01903. 10.1002/cbdv.202501903 41043190

[B9] Cirqueira M. L. Bortot L. O. Bolean M. Aleixo M. A. A. Luccas P. H. Costa-Filho A. J. (2022). Trypanosoma cruzi nitroreductase: structural features and interaction with biological membranes. Int. J. Biol. Macromol. 221, 891–899. 10.1016/j.ijbiomac.2022.09.073 36100001

[B10] Cosar C. Julou L. (1959). The activity of 1-(2-hydroxyethyl)-2-methyl-5-nitroimidazole (R. P. 8823) against experimental trichomonas vaginalis infections. Ann. Inst. Pasteur (Paris). 96, 238–241. 13627590

[B11] Costa F. M. (2018). Planejamento e síntese de novos derivados da associação molecular benzonidazol e metronidazol. Universidade Federal do Pará. Available online at: http://repositorio.ufpa.br:8080/jspui/handle/2011/14492 (Accessed April 12, 2026).

[B12] Daina A. Michielin O. Zoete V. (2017). SwissADME: a free web tool to evaluate pharmacokinetics, drug-likeness and medicinal chemistry friendliness of small molecules. Sci. Rep. 7, 42717. 10.1038/srep42717 28256516 PMC5335600

[B13] de Melo Í. M. Camargo T. P. da Silva V. A. dos Santos E. G. Caldas I. S. Mansur Pontes C. L. (2025). Discovery of a new eugenol-benznidazole hybrid active against different evolutive stages of Trypanosoma cruzi. Bioorg. Chem. 154, 107993. 10.1016/j.bioorg.2024.107993 39591688

[B14] Elgrishi N. Rountree K. J. McCarthy B. D. Rountree E. S. Eisenhart T. T. Dempsey J. L. (2018). A practical beginner’s guide to cyclic voltammetry. J. Chem. Educ. 95, 197–206. 10.1021/acs.jchemed.7b00361

[B15] Goldfarb D. S. (2009). Method for altering the lifespan of eukariotic organisms U.S. patent no WO 2009/086303 A3, 74. Rochester, NY: U.S. World Intellectual Property Organization.

[B16] Gonçalves-Santos E. Caldas I. S. Fernandes V. Â. Franco L. L. Pelozo M. F. Feltrim F. (2023). Pharmacological potential of new metronidazole/eugenol/dihydroeugenol hybrids against Trypanosoma cruzi *in vitro* and *in vivo* . Int. Immunopharmacol. 121, 110416. 10.1016/j.intimp.2023.110416 37295025

[B17] Gosser D. K. J. (1993). “Cyclic Voltammetry—simulation and analysis of reaction mechanisms,” in VCH. 10.1002/elan.1140070324

[B18] Hall B. S. Wilkinson S. R. (2012). Activation of benznidazole by trypanosomal type I nitroreductases results in glyoxal formation. Antimicrob. Agents Chemother. 56, 115–123. 10.1128/AAC.05135-11 22037852 PMC3256028

[B19] Hernández Ceruelos A. Romero-Quezada L. C. Ruvalcaba Ledezma J. C. López Contreras L. (2019). Therapeutic uses of metronidazole and its side effects: an update. Eur. Rev. Med. Pharmacol. Sci. 23, 397–401. 10.26355/eurrev_201901_16788 30657582

[B20] Hood C. A. Fuentes G. Patel H. Page K. Menakuru M. Park J. H. (2008). Fast conventional fmoc solid‐phase peptide synthesis with HCTU. J. Pept. Sci. 14, 97–101. 10.1002/psc.921 17890639

[B47] Jones G. Willett P. Glen R. C. Leach A. R. Taylor R. (1997). Development and validation of a genetic algorithm for flexible docking. J. Mol. Biol. 267 (3), 727–748. 10.1006/jmbi.1996.0897 9126849

[B21] La-Scalea M. A. Serrano S. H. P. Ferreira E. I. Oliveira Brett A. M. (2002). Voltammetric behavior of benznidazole at a DNA-Electrochemical biosensor. J. Pharm. Biomed. Anal. 29, 561–568. 10.1016/S0731-7085(02)00081-X 12062657

[B22] Leitsch D. Schlosser S. Burgess A. Duchêne M. (2012). Nitroimidazole drugs vary in their mode of action in the human parasite giardia lamblia. Int. J. Parasitol. Drugs Drug Resist. 2, 166–170. 10.1016/j.ijpddr.2012.04.002 24533278 PMC3862438

[B23] Lopes M. S. Sales Júnior P. A. Lopes A. G. F. Yoshida M. I. Silva T. H. Romanha A. J. (2011). The activity of a metronidazole analogue and its β-cyclodextrin complex against Trypanosoma cruzi. Mem. Inst. Oswaldo Cruz 106, 1055–1057. 10.1590/S0074-02762011000800027 22241134

[B24] Machado F. P. Campos M. C. Echevarria-Lima J. Sangi D. P. Serpa C. Chaves O. A. (2025). Synthesis of novel tetra-substituted pyrazole derivatives using microwave irradiation and their anti-leukemic activity against jurkat cells. Molecules 30, 2880. 10.3390/molecules30132880 40649394 PMC12251036

[B25] Martins F. M. Sokolovicz Y. C. A. Oliveira M. M. Serpa C. Chaves O. A. Back D. F. (2025). Synthesis, structural characterization, and *in silico* antiviral prediction of novel DyIII-YIII-and EuIII-Pyridoxal helicates. Inorganics 13, 252. 10.3390/inorganics13080252

[B26] Maya J. D. Bollo S. Nuñez-Vergara L. J. Squella J. A. Repetto Y. Morello A. (2003). Trypanosoma cruzi: effect and mode of action of nitroimidazole and nitrofuran derivatives. Biochem. Pharmacol. 65, 999–1006. 10.1016/S0006-2952(02)01663-5 12623132

[B27] Molina I. Salvador F. Sánchez-Montalvá A. Artaza M. A. Moreno R. Perin L. (2017). Pharmacokinetics of benznidazole in healthy volunteers and implications in future clinical trials. Antimicrob. Agents Chemother. 61 (4), e01912–16. 10.1128/AAC.01912-16 28167552 PMC5365666

[B28] Montalbetti C. A. G. N. Falque V. M. (2005). Amide bond formation and peptide coupling. Tetrahedron 61, 10827–10852. 10.1016/j.tet.2005.08.031

[B29] Murteira Pinheiro P. de S. Franco L. S. Montagnoli T. L. Fraga C. A. M. (2024). Molecular hybridization: a powerful tool for multitarget drug discovery. Expert Opin. Drug Discov. 19, 451–470. 10.1080/17460441.2024.2322990 38456452

[B30] Patterson S. Wyllie S. (2014). Nitro drugs for the treatment of trypanosomatid diseases: past, present, and future prospects. Trends Parasitol. 30, 289–298. 10.1016/J.PT.2014.04.003 24776300 PMC4045206

[B31] Pérez-Molina J. A. Molina I. (2018). Chagas disease. Lancet 391, 82–94. 10.1016/S0140-6736(17)31612-4 28673423

[B32] Pitombeira M. C. S. R. Júnior P. A. S. Murta S. M. F. Romanha A. Luccas P. H. Nonato M. C. (2024). New 2‐nitroimidazole‐ N ‐Acylhydrazones, analogs of benznidazole, as anti‐ Trypanosoma cruzi agents. Arch. Pharm. Weinh. 357, 357. 10.1002/ardp.202400059 38627301

[B33] Prasanna S. Doerksen R. (2009). Topological polar surface area: a useful descriptor in 2D-QSAR. Curr. Med. Chem. 16, 21–41. 10.2174/092986709787002817 19149561 PMC7549127

[B34] Ralph E. D. (1983). Clinical pharmacokinetics of metronidazole. Clin. Pharmacokinet. 8, 43–62. 10.2165/00003088-198308010-00003 6340904

[B35] Rocha G. X. de Oliveira A. A. Esteves-Souza A. Serpa C. Chaves O. A. Echevarria A. (2026). Synthesis, *in vitro* anti-sporothrix spp. activity and *in silico* CYP51 interactions of novel regioisomers isatin-thiosemicarbazone hybrids. J. Mol. Struct. 1356, 145074. 10.1016/j.molstruc.2025.145074

[B36] Rodríguez M. A. Mijoba A. Parra-Giménez N. J. Blanco Z. Chávez K. Suárez A. I. (2025). Synthesis, evaluation of the biological activity against Trypanosoma cruzi and leishmania donovani. Preliminary *in silico* ADMET studies of 5-nitroimidazole derivatives. Eur. J. Med. Chem. Rep. 13, 100248. 10.1016/j.ejmcr.2025.100248

[B37] Roney M. Mohd Aluwi M. F. F. (2024). The importance of *in-silico* studies in drug discovery. Intell. Pharm. 2, 578–579. 10.1016/j.ipha.2024.01.010

[B38] Sánches-Pavón E. Márquez-López E. García-Gálvez A. M. Carrera-Huerta F. López-Monteon A. Ramos-Ligonio A. (2012). *In vitro* trypanocidal activity of nitroimidazole derivatives. Lat. Am. J. Pharm. 31, 57–61.

[B39] Shahzad I. Alasmari M. S. Zamir A. Rasool M. F. Alqahtani F. (2025). Clinical pharmacokinetics of metronidazole: a systematic review and meta-analysis. Antimicrob. Agents Chemother. 69, e0190424. 10.1128/aac.01904-24 40741956 PMC12406673

[B40] Shao Y. Molnar L. F. Jung Y. Kussmann J. Ochsenfeld C. Brown S. T. (2006). Advances in methods and algorithms in a modern quantum chemistry program package. Phys. Chem. Chem. Phys. 8, 3172–3191. 10.1039/B517914A 16902710

[B41] Siqueira J. D. Sokolovicz Y. C. A. Oliveira M. M. Martins F. M. Loreto M. F. dos Santos Siqueira F. (2026). High antioxidant and antibiofilm activity of manganese (II and III) complexes derived from pyridoxal and aromatic hydrazides. J. Inorg. Biochem. 276, 113166. 10.1016/j.jinorgbio.2025.113166 41401539

[B42] SwissADME (2025). SwissADME. Available online at: http://www.swissadme.ch/index.php (Accessed April 29, 2025).

[B43] Valeur E. Bradley M. (2009). Amide bond formation: beyond the myth of coupling reagents. Chem. Soc. Rev. 38, 606–631. 10.1039/B701677H 19169468

[B44] Velez A. S. M. M. Chaves O. A. Serpa C. Salem F. M. Li B. Su B. (2026). Contribution to the chemotherapy of human trypanosomiasis: design, synthesis, and biological evaluation of dimeric 2-Nitroimidazoles against Trypanosoma cruzi amastigotes and bloodstream Trypanosoma brucei. ACS Omega 11, 1546–1556. 10.1021/acsomega.5c09284 41552522 PMC12809571

[B45] Yuan S. Chan H. C. S. Hu Z. (2017). Using PyMOL as a platform for computational drug design. WIREs Comput. Mol. Sci. 7, e1298. 10.1002/wcms.1298

