# Supplementary material for: Structure-enabled enhancement of potency in a metronidazole–benznidazole hybrid: design, synthesis, and evaluation of antitrypanosomal activity of a benzylamide-linked 5-nitroimidazole
Source: Front Pharmacol. 2026 Apr 30;17:1812023. doi: 10.3389/fphar.2026.1812023 (PMC13171372; doi:10.3389/fphar.2026.1812023)
Supplement: Supplementary file 1 [file Supplementaryfile1.docx]

**Supplementary Material**

***Structure-Enabled Enhancement of Potency in a Metronidazole–Benznidazole Hybrid: Design, Synthesis, and Evaluation of Antitrypanosomal Activity of a Benzylamide-Linked 5-Nitroimidazole***

**INDEX**

**Figure S1.** Superposition of the obtained ten docking poses to (A) TcNTR:**1**, (B) TcNTR:**2**, and (C) TcNTR:**3** in the presence of coenzyme FMN. Chains A and B, FMN, and the compounds **1, 2,** and **3** are depicted in beige, black, white, pink, cyan, and yellow, respectively. Elements´ color: oxygen, phosphorus, and nitrogen in red, orange, and blue, respectively. For better interpretation, hydrogen atoms were omitted.

**Figure S2.** Pharmacokinetic, physicochemical, and drug-like predictions for **1**.

**Figure S3.** Pharmacokinetic, physicochemical, and drug-like predictions for **2**.

**Figure S4.** Pharmacokinetic, physicochemical, and drug-like predictions for **3**.

**Figure S5.** ^1^H-NMR spectrum of **4** in acetone-*d_6_*.

**Figure S6.** ^13^C-NMR DEPT-135 spectrum of **4** in acetone-*d_6_*.

**Figure S7.** ^1^H-NMR spectrum of **3** in CDCl_3_.

**Figure S8.** ^13^C-NMR DEPT-135 spectrum of **3** in CDCl_3_.

**Figure S9.** HPLC Chromatogram of **3**.

**Figure S10.** HRMS-TOF (MS^+^) of **3**.

**Figure S11.** Inhibition plots of compounds **1-3** against T. cruzi amastigotes at 120 hours.

**Figure S12.** Inhibition plots of compounds **1-3** against LLC-MK2 cells at 120 hours.

**Figure S13.** Cyclic voltammograms of the compounds (a) **1**, (b) **2**, and (c) **3** in 1M KCl solution (pH = 7), with varying the scan rates from 200 to 800 mV/s with solutions under saturated N_2_.

**Figure S14.** Dependence of the potential of the cathodic peak of a 1 mM solution of the compounds **1**, **2**, and **3**, on the logarithm of the scan rate.


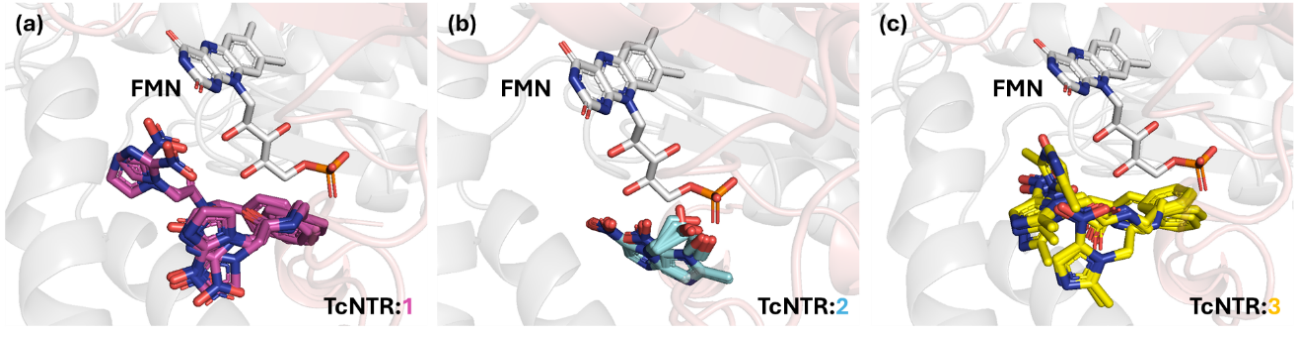


**Figure S1.** Superposition of the obtained ten docking poses to (A) TcNTR:**1**, (B) TcNTR:**2**, and (C) TcNTR:**3** in the presence of coenzyme FMN. Chains A and B, FMN, and the compounds **1, 2,** and **3** are depicted in beige, black, white, pink, cyan, and yellow, respectively. Elements´ color: oxygen, phosphorus, and nitrogen in red, orange, and blue, respectively. For better interpretation, hydrogen atoms were omitted.


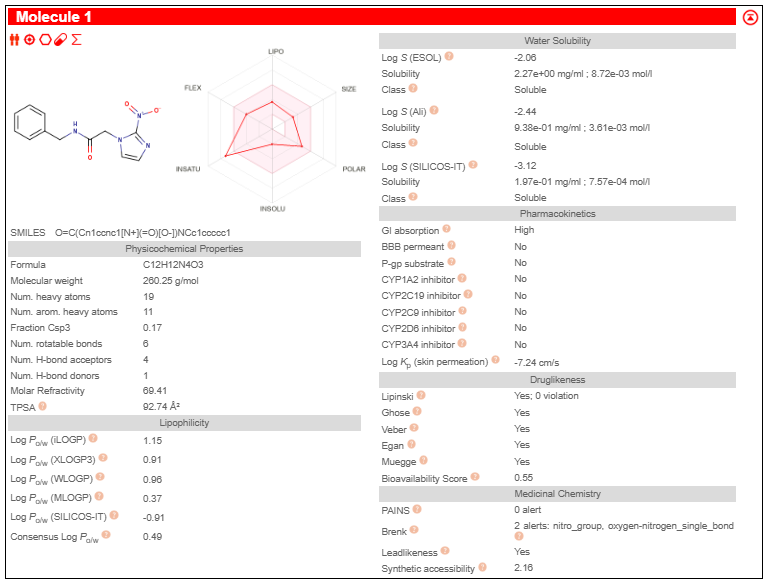


**Figure S2.** Pharmacokinetic, physicochemical, and drug-like predictions for **1**.


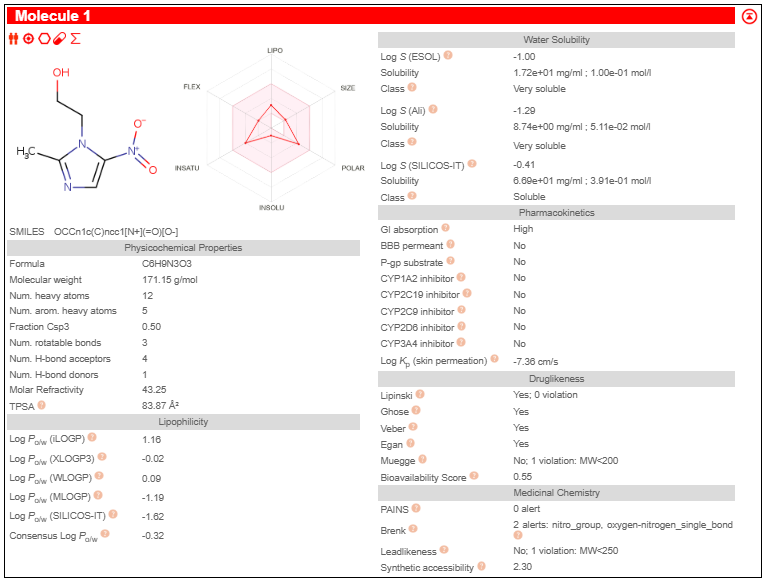


**Figure S3.** Pharmacokinetic, physicochemical, and drug-like predictions for **2**.


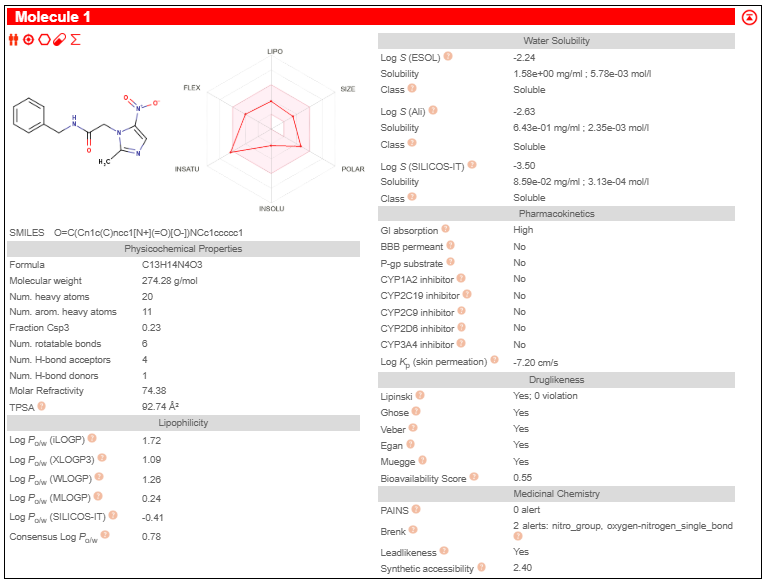


**Figure S4.** Pharmacokinetic, physicochemical, and drug-like predictions for **3**.


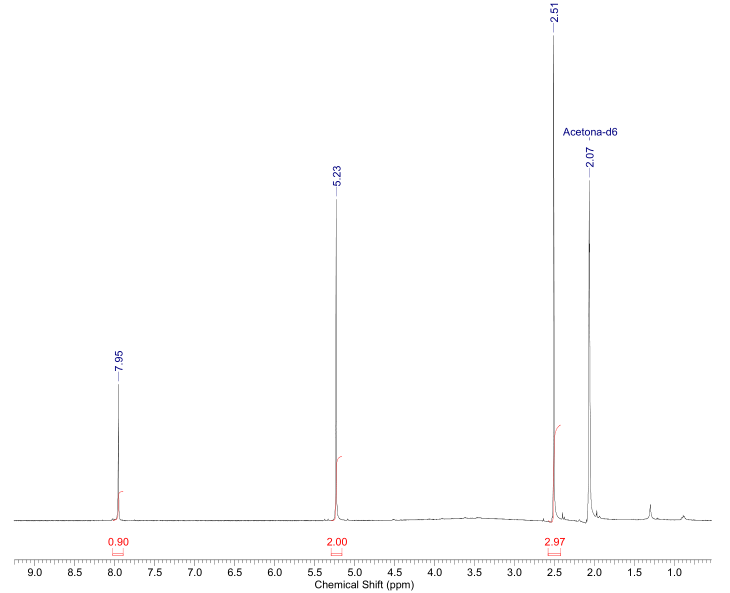


**Figure S5.** ^1^H-NMR spectrum of **4** in acetone-*d_6_*.


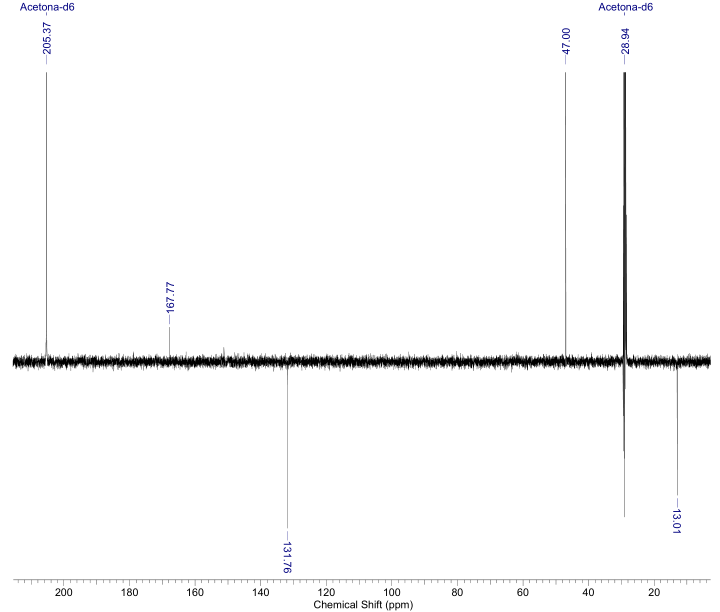


**Figure S6.** ^13^C-NMR DEPT-135 spectrum of **4** in acetone-*d_6_*.


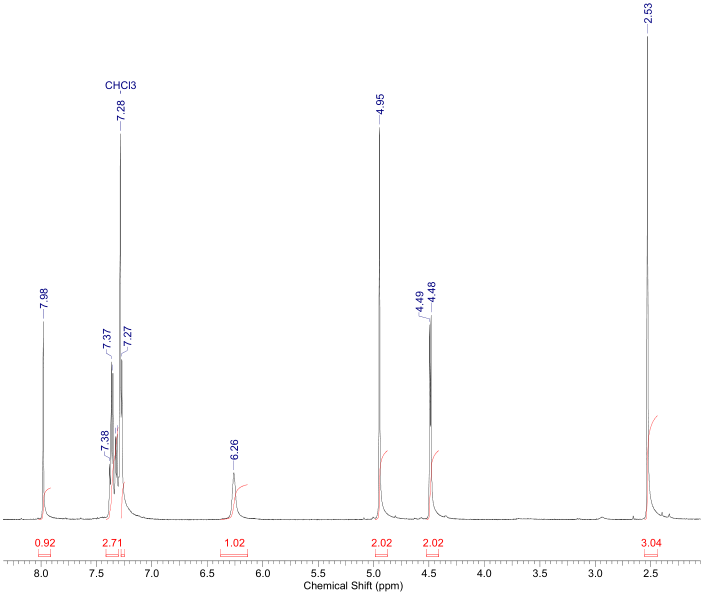


**Figure S7.** ^1^H-NMR spectrum of **3** in CDCl_3_.


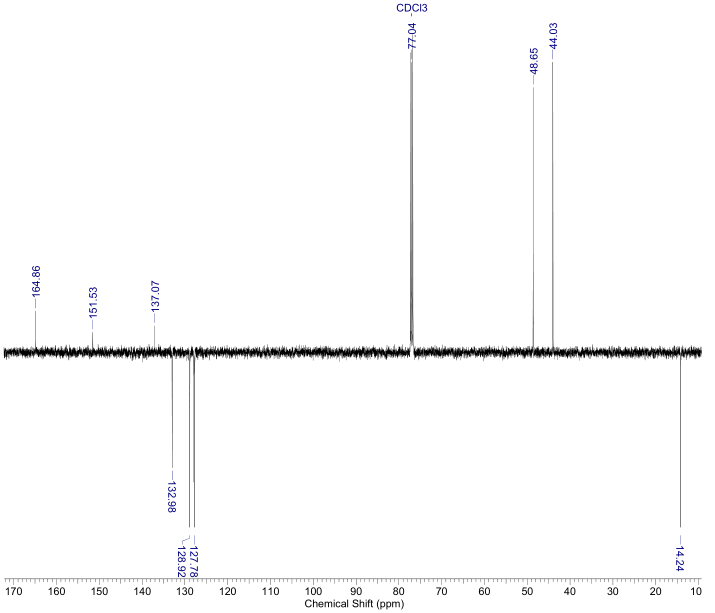


**Figure S8.** ^13^C-NMR DEPT-135 spectrum of **3** in CDCl_3_.


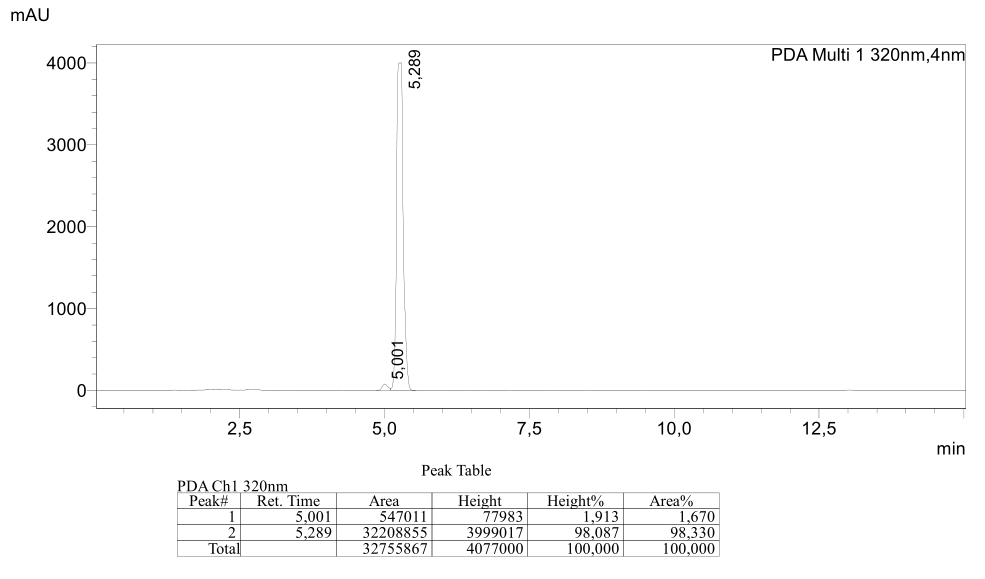


**Figure S9.** HPLC Chromatogram of **3**.


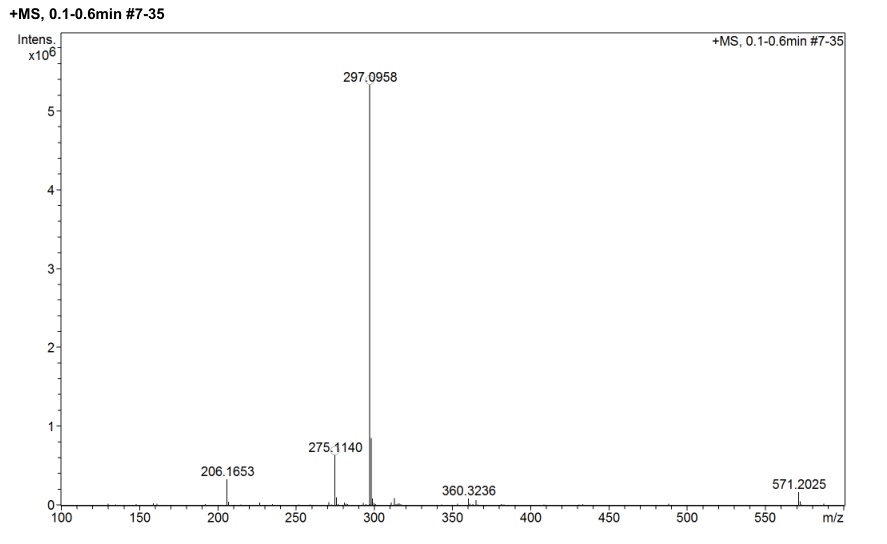


**Figure S10.** HRMS-TOF (MS^+^) of **3**.

**Figure S11.** Inhibition plots of compounds **1-3** against *T. cruzi* amastigotes at 120 hours. The statistically significant differences between treatments are represented by connector lines and ns (non-significant) *(*p* < 0.05), **(*p* < 0.01), ***(*p* < 0.001), **** (*p*<0.0001).

**Figure S12.** Inhibition plots of compounds **1-3** against LLC-MK2 cells at 120 hours. The statistically significant differences between treatments are represented by connector lines and ns (non-significant) *(*p* < 0.05), **(*p* < 0.01), ***(*p* < 0.001), **** (*p*<0.0001).

**Figure S13.** Cyclic voltammograms of the compounds (a) **1**, (b) **2**, and (c) **3** in 1M KCl solution (pH = 7), with varying the scan rates from 200 to 800 mV/s with solutions under saturated N_2_.

**Figure S14.** Dependence of the potential of the cathodic peak of a 1 mM solution of the compounds **1**, **2**, and **3**, on the logarithm of the scan rate.
